# Book Reviews

**Published:** 1893-01

**Authors:** 


### *BOOK REVIEWS.*

---

OVER ONE THOUSAND PRESCRIPTIONS of Various Authors, Teachers and Practicing Physicians. Price $1.00. Illustrated Medical Journal Co., Detroit, Mich.

This book is a collection of the formulæ that have appeared in *Leonard's Illustrated Medical Journal* during the last five years. It presents no advantages over others of its class unless it be that the uses of the latest drugs have been included. A great disadvantage of it is that it makes no effort to classify the diseases, and those who wish to consult the formulæ for any one condition are thus referred possibly to a half-dozen or more diferent pages of the book. The free use of such cut-and-dried formulæ as these is certainly not advisable, but those who wish such a book will find this about as good as any.

---

ADDRESSES AND ESSAYS. By G. Frank Lydston, M. D., Professor of Diseases of the Genito-Urinary Organs and Syphilis in the College of Physicians and Surgeons, Chicago, etc. Second Edition. Renz & Henry, Louisville, Ky.

This is a collection of papers and addresses which Dr. Lydston has read and delivered before different societies within the last few years. The subjects included relate mainly to genito-urinary surgery, in which the author has had rich opportunities for study and observation. Most of the papers have been published before. One of the most interesting, even if not so practical, is the essay on Criminal Crania. The "Treatment of Syphilis," "Gonorrhœa in Women," and "Sexual Perversion" are also valuable papers.

---

A MANUAL OF MEDICAL JURISPRUDENCE AND TOXICOLOGY. By Henry C. Chapman, M. D., Professor of Institutes of Medicine and Medical Jurisprudence in the Jefferson Medical College of Philadelphia, Member of the College of Physicians of Philadelphia, etc. Thirty-six illustrations. Price $1.25. W. B. Saunders, 913 Walnut street, Philadelphia.

This volume of two hundred and forty pages embraces essentially the course of lectures on medical jurisprudence delivered by the author to the students of the Jefferson Medical College during the session of 1891-92. Dr. Chapman's experience for six years as Coroner's physician in Philadelphia has placed him in close relation with this subject, and his book, while by no means exhaustive, is an excellent practical discussion of the principles of medical jurisprudence and embodies all the essential features of this important subject. As an outline for students it achieves its purpose excellently.

We have received from Mrs. Harriet A. Sawyer, 3402 Washington avenue, St. Louis, Mo., a copy of her little volume, "Asheville or the Skyland." This is a very fair description of Asheville, N. C., and the surrounding country, with some poetic license called the "Land of the Sky." Price 75 cents.

---

MOTHER AND CHILD. Part I, *Mother*. By Edward P. Davis, M. D. Part II, *Child*. By John M. Keating, M. D. 472 pages. $2.50. J. B. Lippincott Company, Philadelphia.

The aim of the authors in this book has been "not to supplant the physician, but to supplement his advice and render intelligible those matters that mothers and nurses find difficult to understand." It is not too technical for the popular reader, and if placed in the hands of the mothers of the country it would do a great deal toward replacing the notions and superstitions of knowing old ladies and midwives with scientific care and treatment of the mother and baby. The first part begins with a discussion of those subjects about which girls are rarely properly informed, such as the physiology and hygiene of puberty and womanhood. The greater part of this section is occupied with directions as to the case of the mother before and after confinement.

The section on the child describes the care of the new born infant, the nursing of infants, artificial feeding of infants, etc. The chapters on education and school hygiene deserve to be placed before every public school board in the country. There are also chapters on surgical emergencies, fresh air and exercise, nursing of sick children and the infectious diseases of childhood.

This is a new book, full of sound wisdom and practical ideas for the physician and mother, and we cordially commend it to the careful reading of both.

HISTOLOGY, PATHOLOGY AND BACTERIOLOGY. By Bennett S. Beach, M. D., Lecturer on Histology, Pathology and Bacteriology, New York Polyclinic. 165 pages. $1.00. Philadelphia, Lea Brothers & Co.

DISEASES OF THE EYE, EAR, THROAT AND NOSE. By Frank E. Miller, M. D., Throat Surgeon, Vanderbilt Clinic College of Physicians and Surgeons, New York; James P. McEvoy, M. D., Throat Surgeon, Bellevue Hospital, Out-Patient Department, New York; and John E. Weeks, M D., Lecturer on Ophthalmology and Otology, Bellevue Hospital Medical College, New York. Pocket-size, 12mo., 218 pages, with 89 illustrations and 2 full-page plates. $1.00.

DISEASES OF CHILDREN. By C. Alexander Rhodes, M. D., Instructor in Diseases of Children, New York Post-Graduate Medical School. 160 pages. $1.00. Philadelphia, Lea Brothers & Co.

PHYSIOLOGY. By Frederick A. Manning, M. D., Attending Surgeon Manhattan Hospital, New York. Pocket-size, 12mo., 201 pages, 69 illustrations. $1.00. Philadelphia, Lea Brothers & Co.

These four volumes belong to the Student's Quiz Series, edited by Dr. B. B. Gallaudet, Demonstrator of Anatomy, College of Physicians and Surgeons, New York. So far as quiz compends can meet the needs of medical students these will be found the equal of any. The volumes composing the series have been prepared by gentlemen well qualified for their work, and they appear to have presented the important features of these studies in a very satisfactory manner. While we may therefore cordially recommend these volumes as compends, we would also add the caution that they are not to be exclusively depended upon; that the student must use them only as aids, and that his broad information must be sought in the large standard works.

---

### PAMPHLETS RECEIVED.

We have received from Messrs. P. Blakiston, Son & Co. (1012 Walnut street, Philadelphia) a copy of their Physician's Visiting List for 1893. The fact that the visiting list of the Messrs. Blakiston has reached the forty-second year of its publication is guarantee of its excellence and popularity. This is conveniently arranged for twenty-five patients per week, and contains numerous tables of useful information for ready reference. This makes one of the best visiting lists with which we are acquainted.

